# Humans optimally integrate cutaneous and proprioceptive cues in haptic size perception

**DOI:** 10.3389/fnhum.2025.1653390

**Published:** 2026-01-07

**Authors:** Keon S. Allen, Daniel Goldreich

**Affiliations:** Department of Psychology, Neuroscience and Behaviour, Faculty of Science, McMaster University, Hamilton, ON, Canada

**Keywords:** Bayesian inference, cue combination, computational models, sensory integration, tactile, somatosensory, finger configuration, psychophysics

## Abstract

Sensory perception relies on the brain's integration of multiple imprecise inputs, a process known as cue combination. Previous research has investigated multisensory integration or unisensory integration within non-haptic senses. In contrast, cue combination within the sense of touch has been understudied. Here, we investigated whether humans optimally combine haptic cutaneous and finger configuration cues when discerning the size of a disk held edge-on between the thumb and index fingers. When these two fingers span the disk to contact its perimeter, a finger configuration cue (the perceived distance between the fingers) provides information about the disk's size. Less obviously, cutaneous cues to disk size may result from the indentation of finger pads caused by the disk's curvature. We considered three hypotheses for how humans might use these cues: they might rely solely on the most reliable cue (Winner-Take-All Model, WTA), combine cues based on a simple arithmetic average (Average Model, AVG), or combine cues via an optimal weighted average (Optimal Model, OPT) in which more reliable cues exert proportionately greater influence on the percept. In three experiments involving 34 participants, we compared participant performance to the predictions of the three models. Each experiment tested participants using a two-interval forced-choice (2IFC) paradigm with 3D printed disk stimuli. On each trial, under occluded vision, participants felt two disks sequentially and responded which felt larger. Participants were tested with the cutaneous index finger cue, cutaneous thumb cue, and finger configuration cue individually and with the three cues together. The three experiments were designed to have progressively greater resolution to distinguish the relevant models from one another. In Experiments 1 and 2, the disks were circular. Experiment 3 included non-circular cue-conflict stimuli. The improvement of accuracy in multi-cue conditions, and perceptual effects of cue-conflict stimuli, were broadly consistent with optimal cue combination. Eight of 12 participants were classified as OPT in Experiment 1; eight of 11 in Experiment 2; and 10 of 11 in Experiment 3. The mean confidence of OPT classifications was 0.56, 0.61, and 0.98, respectively. We conclude that humans combine haptic cues optimally to judge the sizes of held objects.

## 1 Introduction

Touch, the first sense to develop in humans (Gallace and Spence, 2010; Gottlieb, 1971), facilitates our interactions with the world and is critical for manipulating, interacting with, and experiencing our environment. Anything felt must be translated from interactions with the skin to patterns of neural impulses that are subsequently decoded by the brain. As the skin is a uniquely movable and deformable organ, the brain presumably incorporates the configuration of the limbs and digits in space, in addition to various cutaneous inputs, in order to interpret the shape of a held object. How does the brain combine these distinct haptic cues in order to achieve a final percept?

Previous cue combination research has generally focused on the integration of cues arising from two or more senses, or on the integration of two or more cues within non-haptic senses. Alais and Burr (2004) found that, when localizing a source that emitted a beep and a flash, participants performed more accurately with both cues than with either alone. Hillis et al. (2004) similarly found that participants more accurately estimated stimulus depth when using two visual cues, texture and binocular disparity, rather than either alone. Crucially, many studies in these domains have found that, during the perceptual process, the brain weights cues according to their reliability (Ernst and Banks, 2002; Hillis et al., 2004; Buckley and Frisby, 1993; Jacobs, 1999; van Beers et al., 2002; Gepshtein and Banks, 2003; Fetsch et al., 2009; Negen et al., 2018), a hallmark of optimal cue combination (Ma et al., 2023; Chapter 5).

In contrast to the well-studied phenomena of multi-sensory and visual cue combination, the integration of cues within the sense of touch has been relatively understudied. In particular, it has been unclear whether human perception during purely haptic exploration conforms to an optimal cue combination strategy (Norman et al., 2004; Klatzky and Lederman, 2011; Hayward, 2008). Here, we investigated whether, and if so, how the brain might combine haptic cues relating to the sizes of different coin-like disks held edge-on between the thumb and index fingers. When feeling for a coin without looking, humans can discern different denominations by their sizes. Clearly, the size of the coin relates to where the fingers are in space through a proprioceptive signal. In order to hold a larger coin, the fingers must be spread farther apart; thus, disk size is presumably signaled at least in part by this finger *Configuration* cue (Config). Less obviously, a coin held on its perimeter between the index finger and thumb exerts pressure that indents the skin of each finger. Given a particular contact force, the indentation profile relates to the size of the coin through its curvature. Smaller coins have greater radii of curvature and produce correspondingly greater skin deformation, which is signaled by the firing rates of slowly adapting tactile afferent axons (Goodwin et al., 1995). We refer to these cutaneous size cues from the thumb and index finger as *Thumb* and *Index* cues, respectively. Do humans combine the *Thumb* cue and *Index* cue with *Config* in order to perceive disk size? If these cues differ in their reliabilities, do humans weight them optimally during the perceptual process?

We investigated three hypotheses for how humans might utilize these haptic cues to perceive the sizes of disk-shaped objects. Perhaps the brain bases its perception exclusively on the most reliable cue and ignores the others. We refer to this possibility as the *WTA* model. Alternatively, perhaps the brain does integrate the three cues but without taking into account their differing reliabilities, instead basing perception on the simple arithmetic average of the cues. We refer to this possibility as the *AVG* model. Finally, perhaps the brain indeed weights each cue according to its reliability, basing perception on an optimal weighted average of the cues. We refer to this possibility as the *OPT* model. All three models produce unbiased perception; that is, over the course of many trials, the mean percept from each model is equal to the value of the stimulus. However, the models differ in their perceptual precision. In particular, the *OPT* model produces the most precise (i.e., less variable) percepts.

Mathematically, the models' distinct perceptual strategies can be represented by the following percept formulae. In these formulae, we denote the stimulus, disk size (mm radius), by *s*, the standard deviation of a cue across repeated trials by σ_*Cue*_, the most reliable cue by Cue*, and the percept (perceived size of the disk) by *ŝ*. For future reference, we define the *reliability* of a cue as equal to the cue's inverse variance, $\frac{1}{\sigma_{Cue}^{2}}$.

*WTA*:


$$\begin{array}{l} \hat{s}=Cue* \end{array}$$ (1)

*AVG*:


$$\begin{array}{l} \hat{s}=\frac{Thumb+Index+Config}{3} \end{array}$$ (2)

*OPT*:


$$\begin{array}{l} \hat{s}=\frac{\frac{Thumb}{\sigma_{Thumb}^{2}}+\frac{Index}{\sigma_{Index}^{2}}+\frac{Config}{\sigma_{Config}^{2}}}{\frac{1}{\sigma_{Thumb}^{2}}+\frac{1}{\sigma_{Index}^{2}}+\frac{1}{\sigma_{Config}^{2}}} \end{array}$$ (3)

With reference to the above percept formulae, note that, when one of the cues is exceptionally reliable (i.e., its sigma is much smaller than the others), the *OPT* and *WTA* models make similar predictions. As the sigma of a cue approaches zero, that cue is increasingly relied upon by an optimal observer, and consequently the *OPT* model reduces to the *WTA* model, where performance is as good as it would be with only the best cue. When the sigmas of the three cues are similar, the *OPT* and *AVG* models give similar predictions, and indeed if the three cues are equally reliable, the *OPT* model reduces to the *AVG* model. In this case, the *OPT* and *AVG* models, because they incorporate the data from all three cues, have better performance than with any one cue. When the cue reliabilities vary, the three models makes different predictions with the *OPT* model yielding the best performance of all. Thus, it is possible to distinguish among the three models, depending on the relative sigmas of the cues.

In three experiments involving a total of 34 participants, we investigated human haptic size perception using a 2-IFC testing protocol. Our goal was to determine the perceptual strategy (e.g., *WTA, AVG*, or *OPT*) that participants used in order to perceive the size of a held disk. In Experiment 1, we presented participants with disks that were designed to be similar in size to coins commonly encountered in daily life, as we reasoned that participants might be able to perceive such disks optimally given their prior experience with coins. In Experiment 2, we shifted the sizes of the disks to be smaller, in an effort to preferentially benefit the cutaneous cues, which were found in Experiment 1 to be much less reliable than the configuration cue. We reasoned that increasing the reliability of the cutaneous cues would allow us to better distinguish the predictions of the *WTA* and *OPT* models. In Experiment 3, we implemented cue conflicts as a further means to create more distinguishing predictions between the models and therefore to increase the confidence with which we could categorize individual participants as either *WTA, AVG*, or *OPT*.

## 2 Methods

Here, we first describe computer simulations and then human experimental methods. We conducted a series of computer-simulated experiments to explore the cue combination behavior that is predicted by each of the three perceptual strategies considered in the study (*OPT, WTA, AVG*), to describe and validate the Bayesian analysis methods that we used for the human data, and to make predictions regarding the informativeness of the human experiments.

### 2.1 Model predictions, computational simulations, and analysis methods

#### 2.1.1 Model predictions

**Figure 1:**
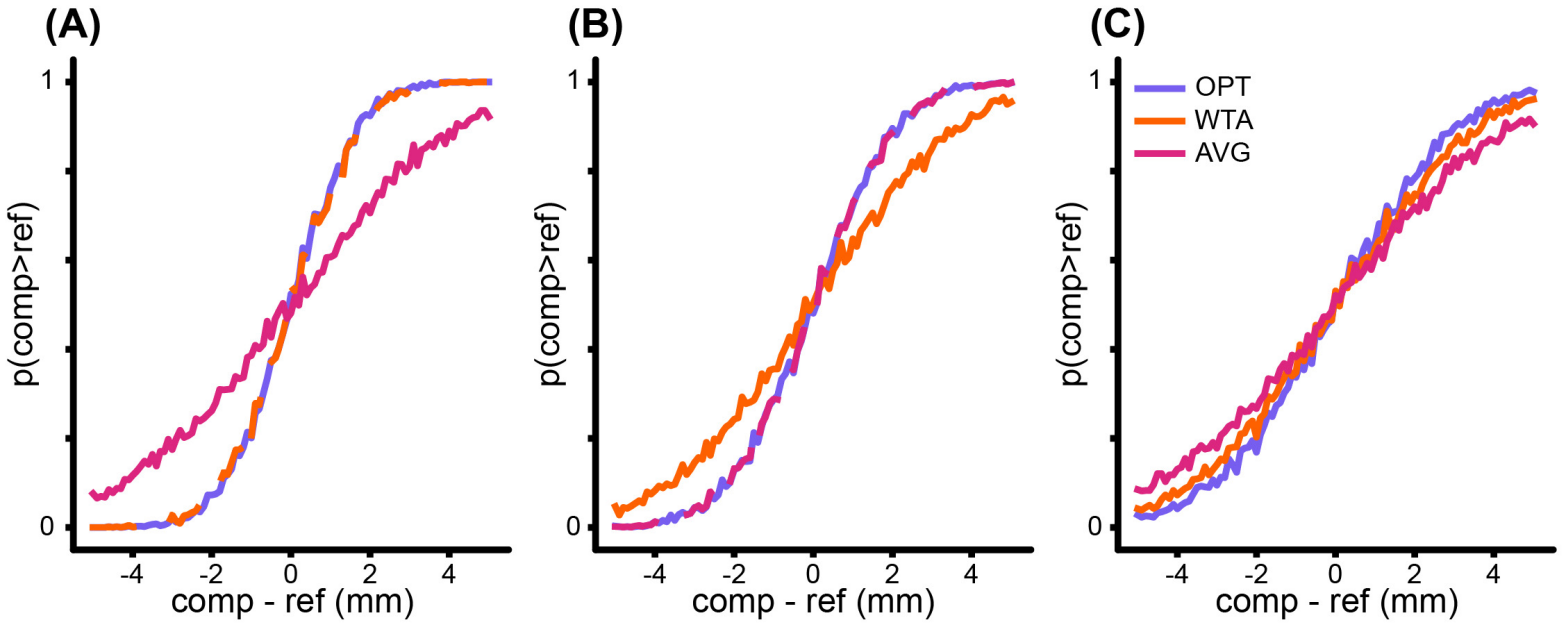
Monte Carlo simulations, from a single simulant, illustrating model predictions under different relative sigma values for the cues. Abscissa: “comp–ref” refers to the size of the comparison disk relative to the reference. Ordinate: “*p*(comp>ref)” refers to the proportion of times the measurement of the comparison was greater than that of the reference. For each of the three models, in each panel, there were 1,000 simulated trials at each 0.1 mm increment from –5 to +5 mm. **(A)** When one cue has a much smaller sigma than the others (i.e., 8, 8, 2 mm), *OPT* and *WTA* models make similar predictions. **(B)** When the cues have similar sigmas (i.e., 4, 4, 4 mm), *OPT* and *AVG* models make similar predictions. **(C)** When the relative sigmas vary (i.e., 7, 6, 3 mm), the models makes different predictions.

We used Monte Carlo simulations to explore the predictions from each model. In a simulated 2-interval forced-choice (2IFC) task, a model participant (simulant) was presented sequentially with two disks of differing size and answered which felt larger. On each trial, one of the disks was a reference disk, while the other was a comparison disk. The size of the comparison disk varied across trials, with a radius from –5 to +5 mm, in increments of 0.1 mm, relative to the fixed reference disk radius. In each interval, when a disk was presented, the model's “nervous system” drew a sensory measurement (cue) from each of three Gaussian distributions centered on the disk radius, with a sigma corresponding to the reliability of the corresponding cue. Each model then processed the three cues according to its particular percept formula (above). On every trial, the simulant reported which of the two disks it perceived to be larger. We simulated 1,000 trials at each comparison level. The proportion of times the comparison disk was judged larger than the reference disk is plotted to create a psychometric function for each model (Figure 1).

Importantly, note that the three models make distinct predictions regarding the variability of the percept (i.e., the standard deviation of the percept across trials), which is reflected in the slope of the corresponding psychometric functions: percepts that are less variable result in steeper psychometric function slopes. From the models' percept formulae (Equations 1, 2, and 3), it is straightforward to derive the standard deviation of the percept across trials when the same disk is felt repeatedly:

*WTA*:


$$\begin{array}{l} \sigma_{\hat{s}}=\sigma_{Cue*} \end{array}$$ (4)

*AVG*:


$$\begin{array}{l} \sigma_{\hat{s}}=\frac{1}{3}\sqrt{\sigma_{Thumb}^{2}+\sigma_{Index}^{2}+\sigma_{Config}^{2}} \end{array}$$ (5)

*OPT*:


$$\begin{array}{l} \sigma_{\hat{s}}=\sqrt{\frac{1}{\frac{1}{\sigma_{Thumb}^{2}}+\frac{1}{\sigma_{Index}^{2}}+\frac{1}{\sigma_{Config}^{2}}}} \end{array}$$ (6)

Note that the *OPT* model percept is less variable than that of the other two models.

#### 2.1.2 Computational simulations

In order to validate our categorization methods and to assess their accuracy, we simulated a realistic Method of Constant Stimuli (MCS) 2-IFC experiment with thousands of simulants who combined cues according to each of the three models. We considered three different populations of simulants defined by the relative sigmas of the sensory cues. Within each population, the sigma for each cue was normally distributed (with mean *M*_*Cue*_ and standard deviation *S*_*Cue*_); to create a simulant, the sigma for each cue was sampled from the corresponding normal distribution.

The three populations were as follows:

Population 1: the sigma of one cue was exceptional (*M*_*Thumb*_ = *M*_*Index*_ = 8*mm, S*_*Thumb*_ = *S*_*Index*_ = 2*mm*; *M*_*Config*_ = 2*mm, S*_*Config*_ = 1*mm*).

Population 2: the sigmas of the three cues were similar (*M*_*Thumb*_ = *M*_*Index*_ = *M*_*Config*_ = 4*mm*; *S*_*Thumb*_ = *S*_*Index*_ = *S*_*Config*_ = 0.1*mm*).

Population 3: the sigmas of the three cues differed (*M*_*Thumb*_ = 7*mm, S*_*Thumb*_ = 2*mm*; *M*_*Index*_ = 6*mm, S*_*Index*_ = 3*mm*; *M*_*Config*_ = 3*mm, S*_*Config*_ = 1*mm*).

The simulated experiment was split into four conditions. In each condition, there were eight comparison levels with 20 trials per comparison level. In the first three conditions, the simulants used each individual cue alone; in the fourth condition, the simulants performed the same task using all three cues together.

On a given trial, the simulant compared a reference stimulus with a comparison stimulus. In the Combined Condition, for example, the measurement distribution for each cue was a Gaussian centered on the true radius of the disk, with a standard deviation equal to the sigma a given simulant had for that cue. In each interval of a trial, the three individual cues were combined according to one of the models (Equations 1, 2, or 3). The simulant determined which interval had the larger percept in order to make its “response” and end a simulated trial. The proportion of times the comparison was perceived to be larger than the reference formed a simulant's psychometric function, which we estimated as a cumulative normal distribution of the stimulus level, Δ (Equation 7).


$$\begin{array}{r} {\text{Ψ}}_{\sigma }\left(\Delta \right)=P\left(comp>ref|\Delta \right)\\ =\int_{0}^{\inf}\frac{1}{\sigma \sqrt{2\pi }}\exp\left(-\frac{{\left(\Delta_{m}-\Delta \right)}^{2}}{2\sigma^{2}}\right)d\Delta_{m} \end{array}$$ (7)

Here, Δ_*m*_ refers to the simulant's internal measurement of the size difference between the two disks presented in a trial, which results from a random sample drawn from a Gaussian distribution centered on the actual size difference (i.e., the comparison disk radius minus the reference disk radius), Δ.

#### 2.1.3 Analysis methods

The analysis methods presented were applied subsequently to the human participant data as well. We used the performance data (D) of each simulant in the three individual-cue conditions to calculate predictions for the simulant's performance in the Combined Condition according to each of the three models. Specifically, for each single-cue condition, for each simulant, we first calculated the likelihood:


$$\begin{array}{l} P\left(D|\sigma_{cue}\right)=\underset{\Delta }{\prod }{\text{Ψ}}_{\sigma }{\left(\Delta \right)}^{k_{\Delta }}{\left(1-{\text{Ψ}}_{\sigma }\left(\Delta \right)\right)}^{n_{\Delta }-k_{\Delta }} \end{array}$$ (8)

where *k*_Δ_ is the number of correct trials and *n*_Δ_ is the total number of trials at Δ, and the psychometric function sigma is equal to σ_*cue*_ $\sqrt{2}$. We then calculated the posterior *PDF* over σ_*cue*_ via Bayes' formula using a uniform prior *PDF*:


$$\begin{array}{l} P\left(\sigma_{cue}|D\right)=\frac{P\left(D|\sigma_{cue}\right)}{\sum_{\sigma_{cue}}P\left(D|\sigma_{cue}\right)} \end{array}$$ (9)

By integrating over the σ_*cue*_ *PDF* for each of the individual-cue conditions, we calculated the σ_*cue*_ *CDFs*. By uniformly sampling from the ordinate of the σ_*cue*_ *CDFs* and interpolating to the abscissa, we sampled sigma values from the σ_*cue*_ *PDFs*. This procedure was preferable to using the mode of the σ_*cue*_ *PDF* as a point estimate because the spread of sampled values reflected the uncertainty of the estimate. A single draw from each of the three σ_*cue*_ *PDFs*, yielded a triplet that served as input values for each of the three model equations (Equations 4–6). Sampling a thousand triplets for each participant, we generated a distribution of Combined Condition sigmas (σ_ŝ_) for each of the three models, for every simulant. We computed the probability of the simulant's performance data given each of these 1,000 model-predicted σ_ŝ_:


$$\begin{array}{l} P\left(D|\sigma_{\hat{s}}\right)=\underset{\Delta }{\prod }{\text{Ψ}}_{\sigma }{\left(\Delta \right)}^{k_{\Delta }}{\left(1-{\text{Ψ}}_{\sigma }\left(\Delta \right)\right)}^{n_{\Delta }-k_{\Delta }} \end{array}$$ (10)

where the psychometric function sigma is equal to σ_ŝ_ $\sqrt{2}$. The average of these 1,000 probabilities implements the marginal likelihood formula for the model, for each participant (Equation 11).


$$\begin{array}{l} P\left(D|M_{i}\right)=\underset{\sigma_{\hat{s}}}{\sum }P\left(D|\sigma_{\hat{s}},M_{i}\right)\cdot P\left(\sigma_{\hat{s}}|M_{i}\right) \end{array}$$ (11)

We categorized the simulant as using the model that had the greatest marginal likelihood. The results from this simulated experiment run on 1,000 simulants per model are summarized in Table 1.

**Table 1:** Simulant classification in a non-conflict experiment.

**Classification**	**OPT simulant**	**WTA simulant**	**AVG simulant**
OPT	**677**	516	98
WTA	315	**445**	250
AVG	8	39	**652**

Bold values indicated correct classifications.

We additionally simulated a cue conflict experiment, in which the reference stimulus in the Combined Condition was no longer a circular disk but rather an oval-like object. The conflict object presented a *Config* cue corresponding to a disk radius that was either larger (positive conflict) or smaller (negative conflict) than the disk radius used to produce the cutaneous cues, *Thumb* and *Index*. In this case, the models make distinct predictions about the mean percept *ŝ*_*con*_ of the conflict object (see Equations 1–3) and consequently the psychometric function incorporates a second parameter, μ, the PSE shift (i.e., difference in percept between the conflict object and the reference object: *ŝ*_*con*_−ŝ_*ref*_; Equation 12):


$$\begin{array}{r} {\text{Ψ}}_{\sigma ,\mu }\left(\Delta \right)=P\left(comp>ref|\Delta \right)\\ =\int_{0}^{\inf}\frac{1}{\sigma \sqrt{2\pi }}\exp\left(-\frac{{\left(\Delta_{m}-\left(\Delta -\mu \right)\right)}^{2}}{2\sigma^{2}}\right)d\Delta_{m} \end{array}$$ (12)

Here, Δ is as defined previously: the size of the comparison disk minus that of the (non-conflict) reference disk. Δ_*m*_ refers to the simulant's internal measurement of the size difference between the two disks presented in a trial (i.e., the comparison disk and the conflict disk), which results from a random sample drawn from a Gaussian distribution centered on the actual size difference, Δ−μ.

Once again, we sampled 1,000 triplets from each simulant's individual cue sigma posterior PDFs. We computed the probability of the simulant's performance data given each model by averaging the 1,000 probabilities of the data given the model-predicted (ŝ, σ_ŝ_) for each triplet. This implements the marginal likelihood calculation for the model (Equation 13):


$$\begin{array}{l} P\left(D|M_{i}\right)=\underset{\hat{s},\sigma_{\hat{s}}}{\sum }P\left(D|\hat{s},\sigma_{\hat{s}},M_{i}\right)\cdot P\left(\hat{s},\sigma_{\hat{s}}|M_{i}\right) \end{array}$$ (13)

**Figure 2:**
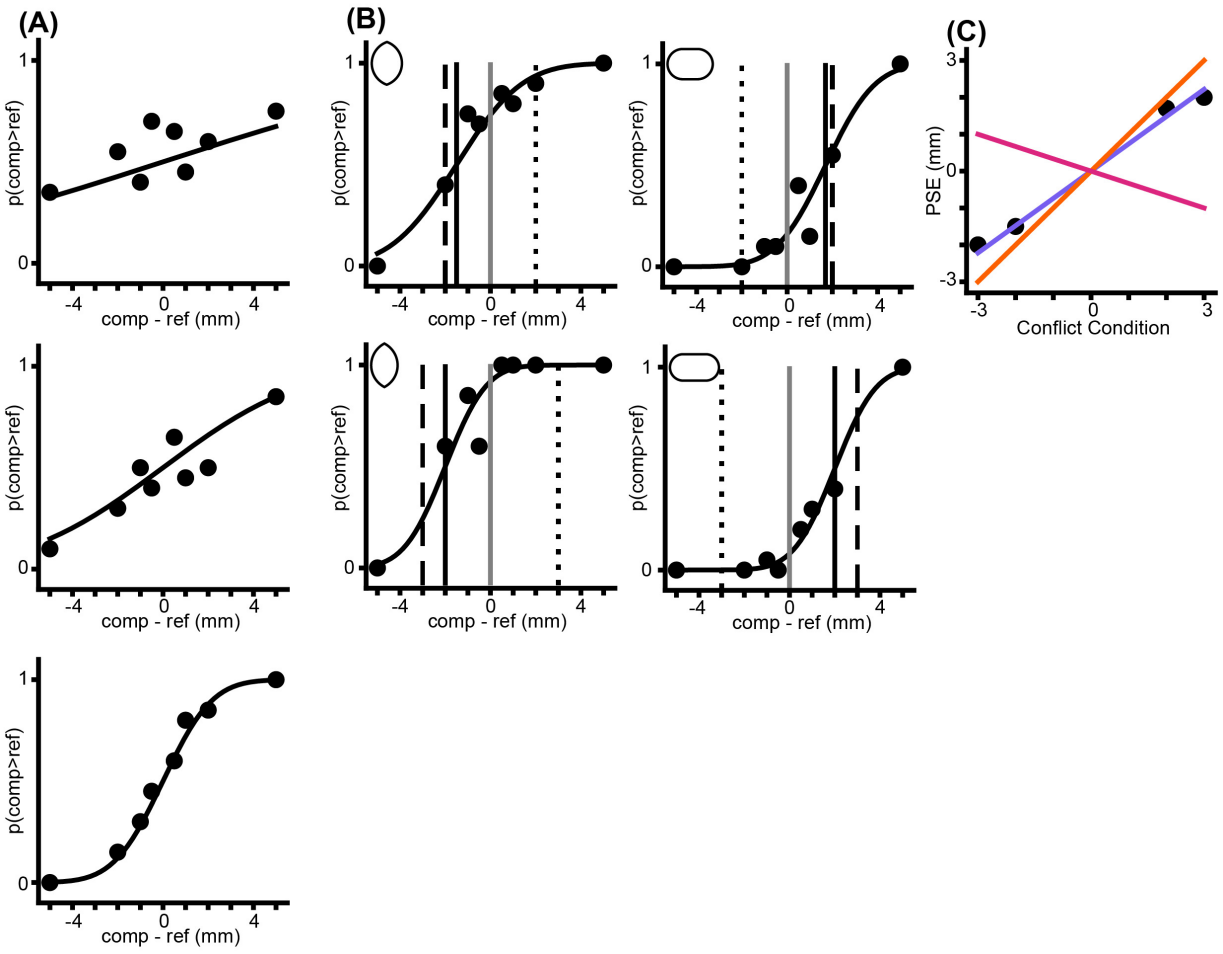
Example of cue-conflict performance from a simulated Bayesian optimal observer with varied sigma. **(A)** Single-cue psychometric functions. From the top: *Thumb, Index, Config*. **(B)** Performance under the four cue conflict conditions (top left: –2, bottom left: –3, top right: +2, bottom right: +3). Black vertical line: *PSE*. Gray line: zero. Dashed line: performance that would result from exclusive reliance on *Config*. Dotted line: performance that would result from exclusive reliance on the two cutaneous cues. **(C)** Plot of *PSE* vs. conflict condition, from **(B)**, along with the predictions of the models, based on the estimated single-cue sigmas from **(A)**. Colors as in Figure 1.

The classifications resulting from runs of 1,000 simulants per model are summarized in Table 2. Figure 2 shows example data from a single *OPT* simulant run on the cue conflict experiment.

**Table 2:** Simulant classification in a cue conflict experiment.

**Classification**	**OPT simulant**	**WTA simulant**	**AVG simulant**
OPT	**870**	157	10
WTA	129	**843**	1
AVG	1	0	**989**

Bold values indicated correct classifications.

These simulations reveal that categorization accuracy was markedly greater for the cue-conflict simulation than for the non-conflict simulations, owing to the distinct predictions that the three models make regarding the percept of the conflict object. We describe below the results of human experiments that support this observation.

### 2.2 Human participants

This study was approved by the McMaster Research Ethics Board. Participants were 34 McMaster University undergraduate students recruited by advertisement in the online SONA system. Persons with any one or more of the following conditions did not participate, as these conditions are known to adversely affect tactile acuity or the ability to perform tactile tasks: diabetes, nervous system disorder or injury (tremor, epilepsy, multiple sclerosis, stroke, etc.), learning disability, dyslexia, attention deficit disorder, cognitive impairment, carpal tunnel syndrome, arthritis of the hands, and hyperhidrosis. Twelve participants took part in Experiment 1 (three women, nine men; mean age 19.3 years; all right-handed). Eleven participants took part in Experiment 2 (one woman, 10 men; mean age 18.8 years; 10 right-handed, one ambidextrous). Eleven participants took part in Experiment 3 (two women, nine men; mean age 19.1 years; eight right-handed, two ambidextrous, one left-handed). Participants gave signed informed consent and received monetary compensation or course credit for their time.

### 2.3 Materials and apparatus

**Figure 3:**
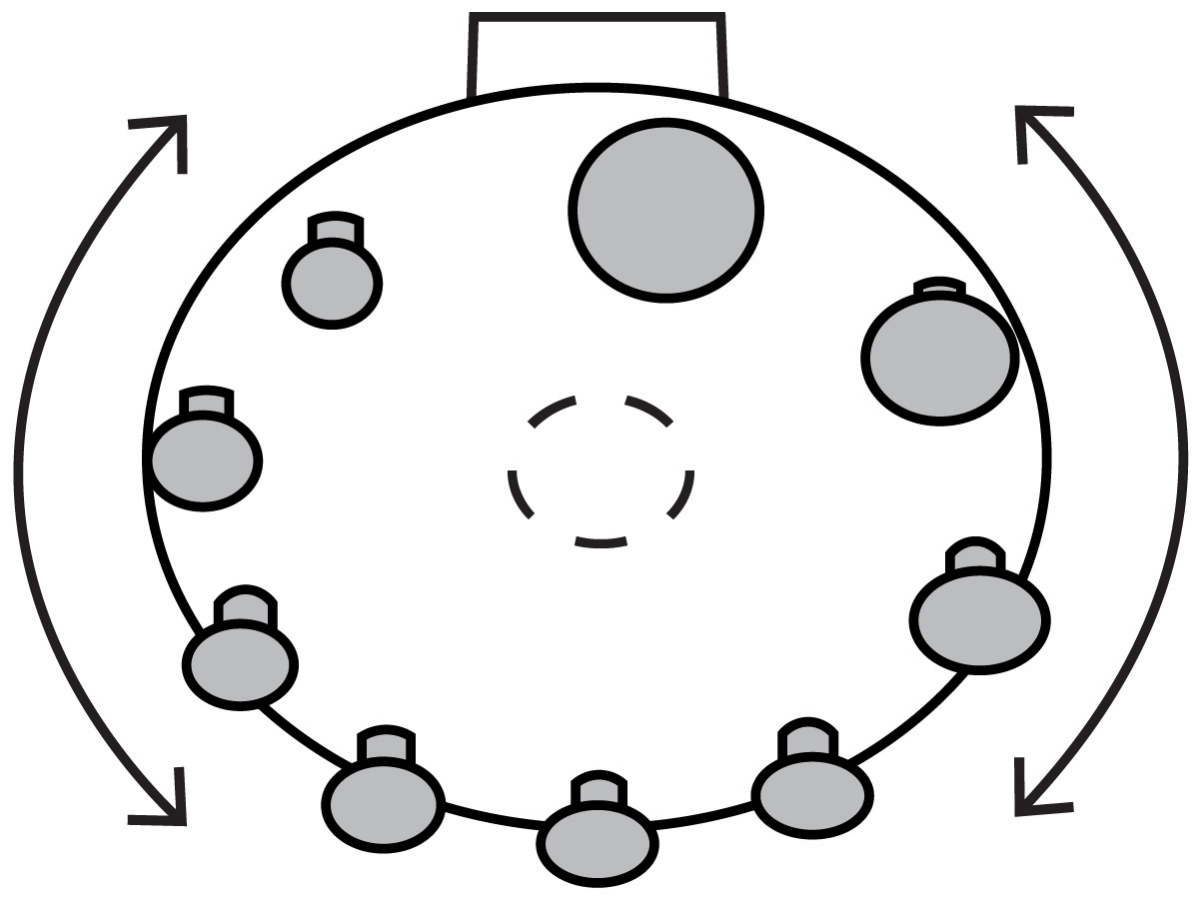
Schematic above-frontal view of the rotating wheel with disk stimuli attached. The wheel is controlled from behind via a stepper motor (not shown). The dashed circle represents the stepper motor axle.

We designed all stimuli in OpenSCAD, a 3D script-based modeler. The stimuli were flat coin-shaped disks of 0.54 mm thickness, 3D printed with PLA plastic using the Ultimaker 2 Go 3D printer. Disk sizes were chosen to be comparable in radius to the size of common coins. For the purposes of psychometric function estimation, disks closer in size to the reference disk (e.g., 15 mm in Experiment 1 or 10 mm in Experiments 2 and 3; see Procedures below) are more informative than the disks farther in size from the reference. Thus, the use of unequal increments in disk size (i.e., step size from one disk to the next increasing as a function of separation from the reference disk size) was preferable given practical limitations on the possible number of disks and experiment time. Accordingly, disks in Experiment 1 were circular with the following radii in mm: 10, 13, 14, 14.5, 15, 15.5, 16, 17, and 20. In Experiments 2 and 3, the disk sizes were reduced, as the results of Experiment 1 indicated that the configuration cue was much more reliable (smaller sigma) than the cutaneous cues, potentially hindering our ability to distinguish the predictions of the *OPT* and *WTA* models. We predicted that smaller disks might lead to more reliable cutaneous cues (Goodwin et al., 1995) while leaving the configuration cue relatively unaffected. Accordingly, disks in Experiments 2 and 3 were circular with the following radii in mm: 5, 8, 9, 9.5, 10, 10.5, 11, 12, and 15. Experiment 3 additionally made use of a set of conflict disks with altered shape. In each experiment, disks were fastened to a wheel that rotated to present disks of varying radii for a given trial (Figure 3). This wheel was controlled by an ISM-7411 NEMA 23 National Instruments Integrated Stepper motor that interfaced with the computer via an Ethernet cable. The computer ran a custom LabVIEW experiment program that used the LabVIEW SoftMotion module to implement stepper motor commands. Plastic thimbles, used in *Config* trials (see below), were 0.5 mm in thickness, designed and 3D printed using the above resources, in a range of sizes such that each participant found two thimbles that fit comfortably over their index finger and thumb. A 3D printed hand rest allowed the thumb and index finger to protrude forward in horizontal alignment while the remainder of the hand was held comfortably stable. The hand rest sat upon an adjustable laboratory scissor jack which acted as a rest and guide for the hand. The jack was affixed to a Heavy Duty 20-inch linear bearing slide rail (Firgelli Automations Canada). The rail allowed participants to slide their hands to the appropriate disk as their arms extended. An FE7B series photoelectric retroreflective infrared light sensor (Honeywell) sent a voltage change to a USB 6210 data acquisition device (National Instruments) when its beam was broken or unbroken by participant movement during trial presentations. Thus, the computer was aware of hand positioning relative to the wheel. The disks, wheel, motor, and other equipment were hidden from participant view behind a foam core box. Participant responses were recorded with a Kensington wireless Bluetooth clicker, on which the participant pressed one of two response keys with the left hand; the clicker's responses were received and recorded by a custom-written computer program that also controlled the experiment apparatus. This computer program was coded in National Instruments LabVIEW 2018 on PC.

### 2.4 Psychophysical procedures

Each experiment used a *2IFC* paradigm in order to test participants' ability to discern which of two disks, felt sequentially, had a larger diameter. The right hand was oriented such that the index finger and thumb were in the horizontal plane. The participants flexed their index finger and/or thumb at the metacarpophalangeal joint in order to touch a disk. On a given trial, a beep signaled the participant to slide their right arm forward to feel the presented target disk. Arm movement was signaled to the computer program by the obstruction of the infrared beam. A second beep, occurring 1,000 ms after beam break, signaled the participant to slide their hand back to the initial position. The computer then rotated the stepper motor in order to select a different target disk for the participant to feel. A third beep then signaled the participant to again slide their arm forward and feel this second disk. A final, higher-pitched beep, occurring 1,000 ms after beam break, signaled the participant to slide their hand back to the initial rest position. The participant then responded which disk felt larger, the first or the second, by pressing one of two response buttons with the Bluetooth clicker in their left hand.

On each trial, one of the two presented disks was a reference disk (presented in every trial) and the other was a comparison disk. The order of presentation of the reference and comparison disks was randomized by the computer program (the program used a random number generator to select either order with 0.5 probability). Participants completed up to thirty practice trials with feedback before starting each condition.

We implemented controls for timing and distance cues. In order to present a particular target disk, the computer first rotated the wheel to a random disk position, preventing the participant from using timing cues to inform their response. During single-digit experiment blocks, the wheel was calibrated to rotate such that the outer edge of each disk was aligned to be the same distance from the participant's digit. Thus, the participant could not benefit from proprioceptive or timing cues associated with the flexion of the finger toward the disk. During two-digit blocks, the wheel was calibrated to keep all presented disks centered at the same position.

**Figure 4:**
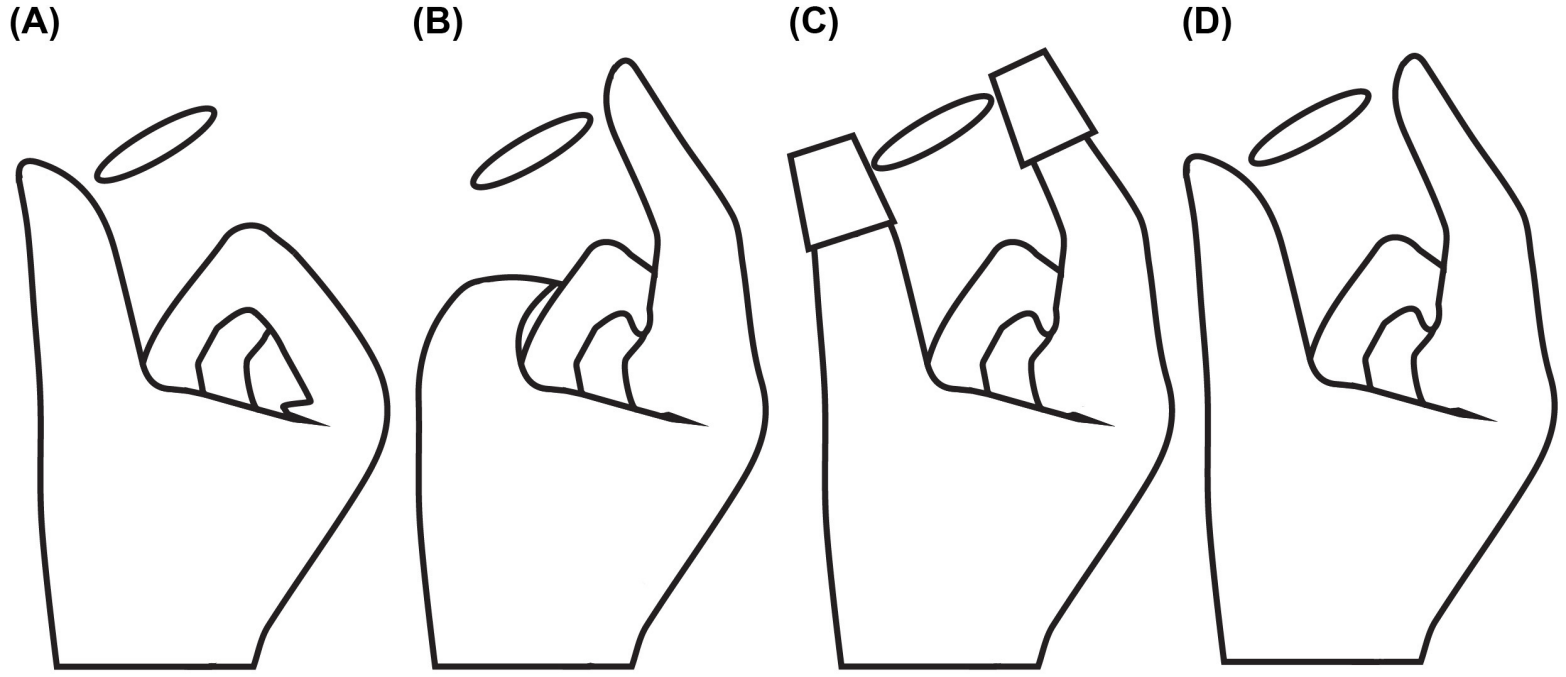
Demonstration of the cue conditions. **(A)** *Thumb* (cutaneous cue on the thumb). **(B)** *Index* (cutaneous cue on the index). **(C)** *Config* (finger configuration cue). **(D)** *Combined* (all cues present).

Participants in Experiments 1 and 2 were tested in four different conditions measuring performance when they had access to the cues alone and all together. The four conditions were as follows: the *Thumb* condition, in which participants had access to the cutaneous cue on the thumb; the *Index* condition, in which participants had access to the cutaneous cue on the index finger; the *Config* condition, in which participants had access to the finger configuration cue; and the *Combined* condition, in which participants had access to all cues together (Figure 4). In the *Thumb* and *Index* conditions, participants used only the thumb or index finger, respectively, of their right hand to feel the lateral edge perpendicular to the face of the disk. The disks were left-aligned for the thumb and right-aligned for the index such that the time and distance required by the participant to contact the disk by flexion of the finger, when the hand was in place, did not vary systematically with disk size. In the *Config* condition, participants wore hard plastic thimbles on their index finger and thumb. Thus, as they felt each disk they had access to the *Config* cue but, because the disk was blocked by the thimbles from indenting their skin, they did not have access to either the *Thumb* cue or *Index* cue. In the *Combined* condition, participants did the same task with thimbles removed, thereby having access to all three cues: *Thumb, Index*, and *Config*. In order to maintain participant concentration, testing for all experiments took place in blocks of 40-trials each, with a minimum rest period of 1 min between blocks, and a 5-min rest period approximately halfway through the testing session (i.e., after block 4 in Experiments 1 and 2, and after block 3 in Experiment 3).

#### 2.4.1 Experiment 1

Experiment 1 took place over two sensory testing sessions of approximately 2.5 h each, spaced one week apart. During each session, participants completed two consecutive 40-trial blocks of each condition. Accordingly, each participant completed a total of 2 days × 4 conditions × 80 trials = 640 trials. The order of conditions was partially counterbalanced across participants, such that the Cutaneous (*Thumb* or *Index*), *Config*, and *Combined* conditions occurred with equal frequency at the beginning, middle, or final positions. Specifically, the orders of conditions tested for the participants were randomly chosen, without replacement, from the twelve possible orders shown in Table 3. For a given participant, the same order of conditions was used in each of the two sensory testing sessions. Disks in Experiment 1 were circular with the following radii in mm: 10, 13, 14, 14.5, 15, 15.5, 16, 17, and 20. The 15 mm disk was the reference to which any of the other disks was compared during each trial. Each testing block was conducted via a Method of Constant Stimuli, with each of the eight comparison disks being delivered five times in pseudo-random order.

**Table 3:** Counterbalanced condition orders in Experiment 1.

1.	Index	Thumb	Config	Combined
2.	Index	Thumb	Combined	Config
3.	Combined	Index	Thumb	Config
4.	Config	Index	Thumb	Combined
5.	Combined	Config	Index	Thumb
6.	Config	Combined	Index	Thumb
7.	Thumb	Index	Config	Combined
8.	Thumb	Index	Combined	Config
9.	Combined	Thumb	Index	Config
10.	Config	Thumb	Index	Combined
11.	Combined	Config	Thumb	Index
12.	Config	Combined	Thumb	Index

#### 2.4.2 Experiment 2

Experiment 2 took place in one sensory testing session of approximately 2.5 h, consisting of two consecutive 40-trial blocks of each condition. Accordingly, each participant completed a total of 4 conditions × 80 trials = 320 trials. The order of conditions was counterbalanced across participants in the same way as in Experiment 1. Disks in Experiment 2 were circular with the following radii in mm: 5, 8, 9, 9.5, 10, 10.5, 11, 12, and 15. The 10 mm disk was the reference to which any of the other disks was compared during each trial. For each condition, sensory testing was conducted via a Bayesian Adaptive Procedure, which selected the comparison disk to present on each trial in order to maximize the expected information gain regarding the participant's psychometric function (Kontsevich and Tyler, 1999; Goldreich et al., 2009). Owing to the efficiency of the Bayesian adaptive procedure, only one sensory testing session was needed to obtain accurate estimates of a participant's psychometric functions, unlike with the method of constant stimuli used in Experiment 1, for which a greater number of trials were required. The Bayesian adaptive procedure can be applied to any psychometric function parameterization; we defined our psychometric functions as cumulative normal distributions of the stimulus level, Δ (Equation 7).

#### 2.4.3 Experiment 3

Experiment 3 took place in one sensory testing session of approximately 2.5 h. Comparison disks in Experiment 3 were circular with the following radii in mm: 5, 8, 9, 9.5, 10.5, 11, 12, and 15. The methodology used was the same as in Experiment 2 with the following exceptions. Instead of two blocks of 40 trials for each of 4 conditions, participants completed one block of 40 trials for each of 7 conditions (a total of 280 trials). The first three blocks completed by participants consisted of individual cue conditions (*Thumb* cue, *Index* cue, and *Config* cue), with a circular reference disk of 10 mm radius, as in Experiment 2; the remaining four blocks consisted of *Cue Conflict* conditions, in which a *Cue Conflict* stimulus served as the reference.

**Figure 5:**
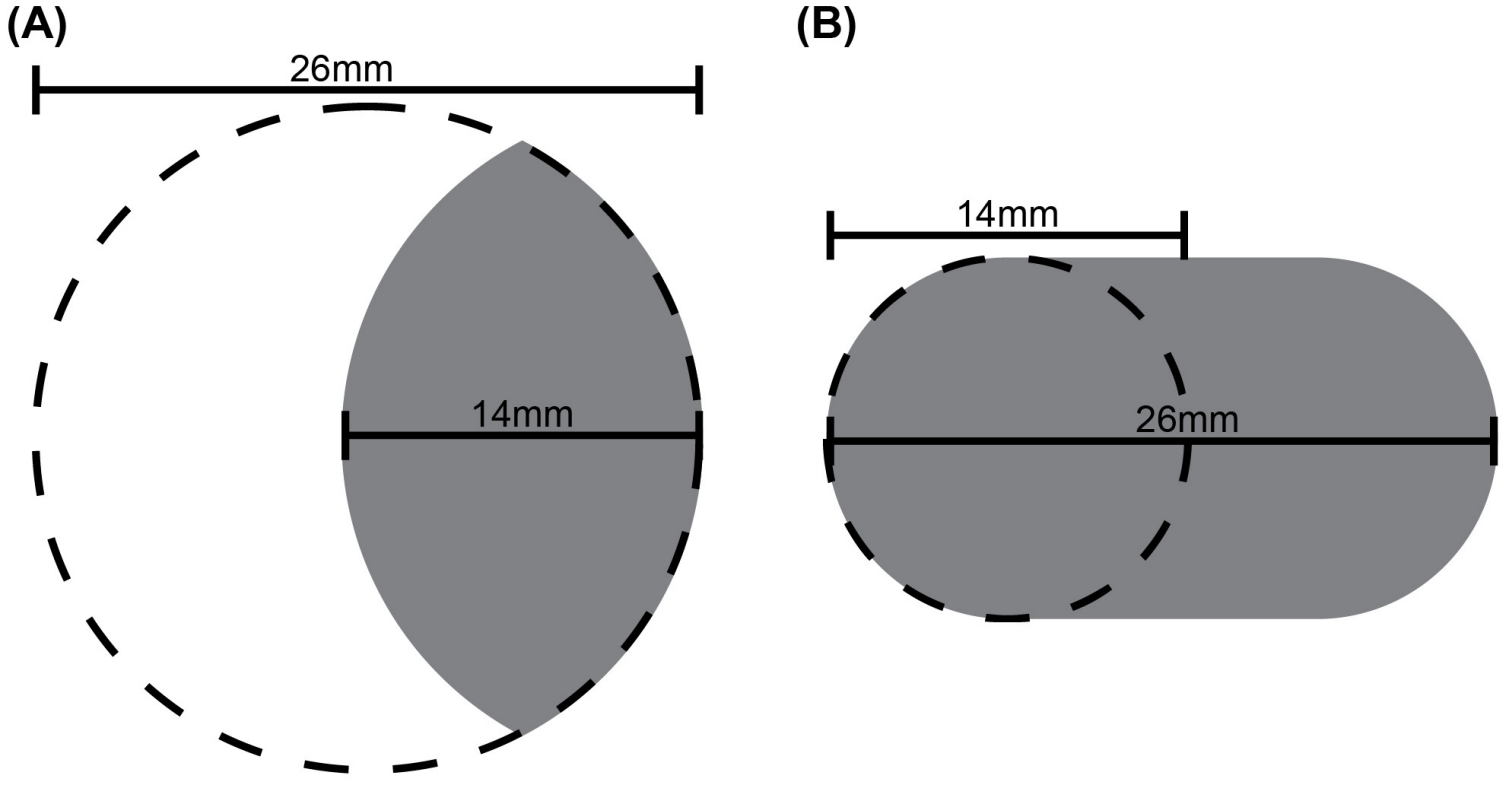
Illustration of two of the four conflict stimuli designed to dissociate cutaneous and proprioceptive cues. Recall that participants felt disks laterally along the horizontal axis. **(A)** The –3 conflict stimulus (shaded) has a horizontal extent equivalent to that of a –3 mm radius disk, and a curvature equivalent to that of a +3 mm radius disk, relative to a 10 mm radius circular disk. **(B)** The +3 conflict stimulus (shaded) has a horizontal extent equivalent to that of a +3 mm radius disk, and a curvature equivalent to that of a –3 mm radius disk, relative to a 10 mm radius circular disk.

The *Cue Conflict* stimuli were designed to dissociate cutaneous and proprioceptive cues. These stimulus pieces were not circular but rather, unknown to the participant, were oval-like. They were 3D printed with a composite design, such that each conflict stimulus had a curvature equal to that of a circular disk with a particular radius, and a width equal to that of a circular disk with a different radius. We used four conflict stimuli, each identified by a signed number. The magnitude of the number indicates the difference in mm between the horizontal half-width of the conflict stimulus and the radius of the (non-conflict, circular) reference disk, while the sign indicates the direction (smaller or larger) of this difference. For instance, the **–3** conflict stimulus (Figure 5A) has a horizontal half-width of 7 mm, which is 3 mm less than the 10 mm radius of the reference disk. Simultaneously, this conflict stimulus has the lateral curvature of a disk of 13 mm radius, which is 3 mm greater than the 10 mm radius of the reference disk. The **+3** conflict disk (Figure 5B), in contrast, has a horizontal half-width of 13 mm, which is 3 mm greater than the 10 mm radius of the reference disk. Simultaneously, this conflict stimulus has the lateral curvature of a disk of 7 mm radius, which is 3 mm less than the 10 mm radius of the reference disk. The same logic applies to the **–2** and **+2** conflict disks.

From the participant's point of view, the *Cue Conflict* conditions were no different from the *Combined* conditions of the earlier experiments. The participant's task was to compare each of the two (assumed circular) disks presented on any trial and report which felt larger. Unknown to the participant, however, the 10 mm radius circular reference disk was swapped out for one of the four different cue conflict stimuli. Accordingly, on each trial, only the comparison disk was actually circular.

Testing conditions were partially counterbalanced across participants The first three blocks were individual cue conditions, which were counterbalanced into the following possible orders: *Config, Thumb, Index*; *Config, Index, Thumb*; *Thumb, Index, Config*; or *Index, Thumb, Config*. The following 4 blocks were conflict conditions. These were also counterbalanced, with the ±**2** conditions occurring sequentially (order counterbalanced across participants), and similarly for the ±**3** conditions. Thus, possible orders of the conflict conditions for a participant were [−2, +2, −3, +3], [+2, −2, +3, −3], [−3, +3, −2, +2], or [+3, −3, +2, −2].

### 2.5 Statistical analyses of human data

For each condition, each participant's performance data were pooled across testing blocks and (for Experiment 1) testing sessions for subsequent analysis.

For Bayesian parameter estimation, we discretized sigma in steps of 0.05 mm from 0.01 mm to 40.01 mm and applied a uniform prior. We parameterized each participant's psychometric function (Equation 7) and estimated sigma as described (Equations 8, 9). To estimate each participant's PSE shift from their cue conflict data, we additionally applied a uniform prior to mu from –5 to +5 mm in 0.1 mm increments, and calculated a joint posterior PDF for the participant's (mu, sigma; see Equation 12), starting with a uniform prior. We then marginalized the joint posterior over sigma to obtain the posterior PDF for mu, and took its mode as the PSE shift estimate.

For frequentist analyses, the mode of each participant's posterior PDF over sigma was taken as a point estimate. We used one-way repeated measures ANOVAs to compare sigma across experimental conditions and separate one-way repeated measures ANOVAs to compare predicted sigma values across the models. Significant ANOVA results were further explored with *post-hoc* paired-samples *t*-tests and Cohen's *d* effect size estimates. Each participant's individual-cue sigmas were combined according to Equations 4–6 to calculate each model's prediction for the participant's combined-cue sigma. Frequentist statistical analyses were completed using Python and relevant packages and modules including pandas, numpy, statsmodels, and matplotlib.

For Bayesian model comparison, we calculated the probability that individual participants were conforming to either the *OPT, AVG*, or *WTA* model, with the marginal likelihood formulas given by Equations 11, 13. From these marginal likelihoods, and using a uniform prior for the models, we calculated the posterior probability of each model for each participant. For clarity of interpretation, we prefer to report posterior probabilities for the models, rather than Bayes Factors. However, as we used uniform prior probabilities for the models, we note that the reader may readily convert the reported posterior probabilities into Bayes factors comparing any two models by dividing the corresponding pair of posterior probabilities.

## 3 Results

We conducted three experiments with a total 34 participants in order to assess the reliability with which people could distinguish disk size based on individual haptic cues involving skin indentation and finger configuration (*Thumb, Index, Config*) and with the three cues combined. Experiments 1 and 2 used circular disks, with the goal of determining whether performance improved when participants had access to more cues, a hallmark of optimal cue combination. Experiment 3 additionally incorporated non-circular conflict stimuli, in order to further distinguish the perceptual strategy used by the participants.

### 3.1 Experiment 1

**Figure 6:**
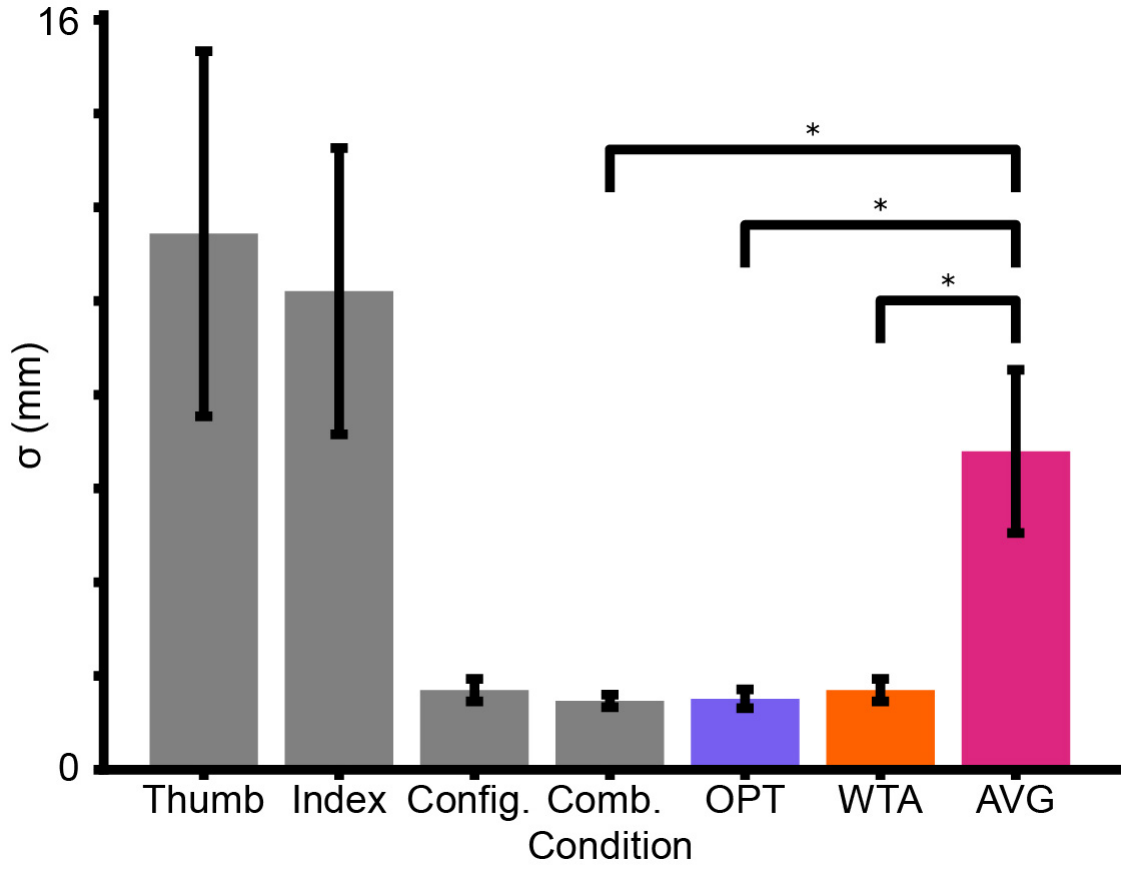
Mean sigma estimates across participants in Experiment 1 (gray bars). Mean model predictions (colored bars) for performance in the Combined Condition, based on the *Thumb, Index*, and *Config* Conditions. Error bars = ± SE. Asterisk (*) = Significant *t*-test.

In Experiment 1, participants compared the sizes of two sequentially presented disks. In different conditions, the participants had access to either a single sensory cue or to the three cues combined. Figure 6 plots the mean single-cue and combined-cue sigma estimates across participants, along with the combined-cue sigma predicted by each model on the basis of the single-cue sigma values. The plot clearly indicates that reliability was higher (i.e., sigma was lower) for the configuration cue than for either of the cutaneous cues. Furthermore, the Combined Condition had a reliability that was similar to that of the configuration condition.

One-way repeated measures ANOVAs revealed significant effects of condition [*p* = 0.006, *F*_(3, 33)_ = 4.961] and model prediction [*p* = 0.001, *F*_(2, 22)_ = 9.038]. Paired-sample *t*-tests demonstrate that the *AVG* model prediction differed significantly from both the *OPT* [*p* = 0.010, *t*_(11)_ = 3.094, Cohen's *d* = 1.231] and *WTA* model predictions [*p* = 0.013, *t*_(11)_ = 2.922, Cohen's *d* = 1.184]. There was, however, no significant difference between the predictions of the *WTA* and *OPT* models [*p* = 0.050, *t*_(11)_ = 2.197]. Importantly, there was a significant difference between the Combined Condition and the *AVG* model prediction [*p* = 0.010, *t*_(11)_ = 3.099, Cohen's *d* = 1.245] but not between Combined and *OPT* [*p* = 0.730, *t*_(11)_ = 0.354] or Combined and *WTA* [*p* = 0.206, *t*_(11)_ = 1.343]. These results indicate convincingly that participants were not in general using an *AVG* perceptual strategy but leave open the possibility that they were using either *WTA* or *OPT*. Intriguingly, close inspection of Figure 6 reveals that the mean performance of participants in the Combined Condition was nearly identical to the *OPT* model prediction and somewhat better (though not significantly so) than the *WTA* model prediction.

For a higher-resolution, single-participant analysis, we conducted Bayesian model comparison in order to calculate posterior probabilities for each participant for each model. The individual posterior probabilities for each participant in Experiment 1 are reported in Table 4. Bayesian model comparison categorized eight out of 12 participants as *OPT*, 3 as *WTA*, and 1 as *AVG*. For the eight participants categorized as *OPT*, the mean posterior probabilities of *OPT* and of *WTA* were 0.56 and 0.37, respectively, indicating substantial uncertainty in the classification.

**Table 4:** Experiment 1 model posterior probabilities by participant.

**Participant**	**OPT**	**WTA**	**AVG**	**Classification**
1	0.472	0.527	0.001	WTA
2	0.458	0.540	0.002	WTA
3	0.507	0.493	0.000	OPT
4	0.535	0.463	0.002	OPT
5	0.548	0.447	0.004	OPT
6	0.052	0.182	0.766	AVG
7	0.588	0.045	0.367	OPT
8	0.481	0.519	0.000	WTA
9	0.548	0.451	0.000	OPT
10	0.560	0.440	0.000	OPT
11	0.665	0.118	0.217	OPT
12	0.528	0.472	0.000	OPT

### 3.2 Experiment 2

Experiment 1 provided evidence against the *AVG* model but was unable to convincingly distinguish between the *OPT* and *WTA* models. We reasoned that this difficulty may have resulted from the fact that *Config* had a much smaller sigma than *Thumb* and *Index* in most participants. When one cue is much more reliable than the others, the predictions of the *OPT* and *WTA* models converge, making it difficult to distinguish between them (see Equations 4, 6). In Experiment 2, with the goal of enhancing *Thumb* cue and *Index* cue, we made the disks smaller so that they would indent the fingers more sharply. We hoped thereby to reduce the sigma of the cutaneous cues so that the cutaneous and configuration cues would have more similar sigma values, facilitating the classification of participants as either *OPT* or *WTA*.

**Figure 7:**
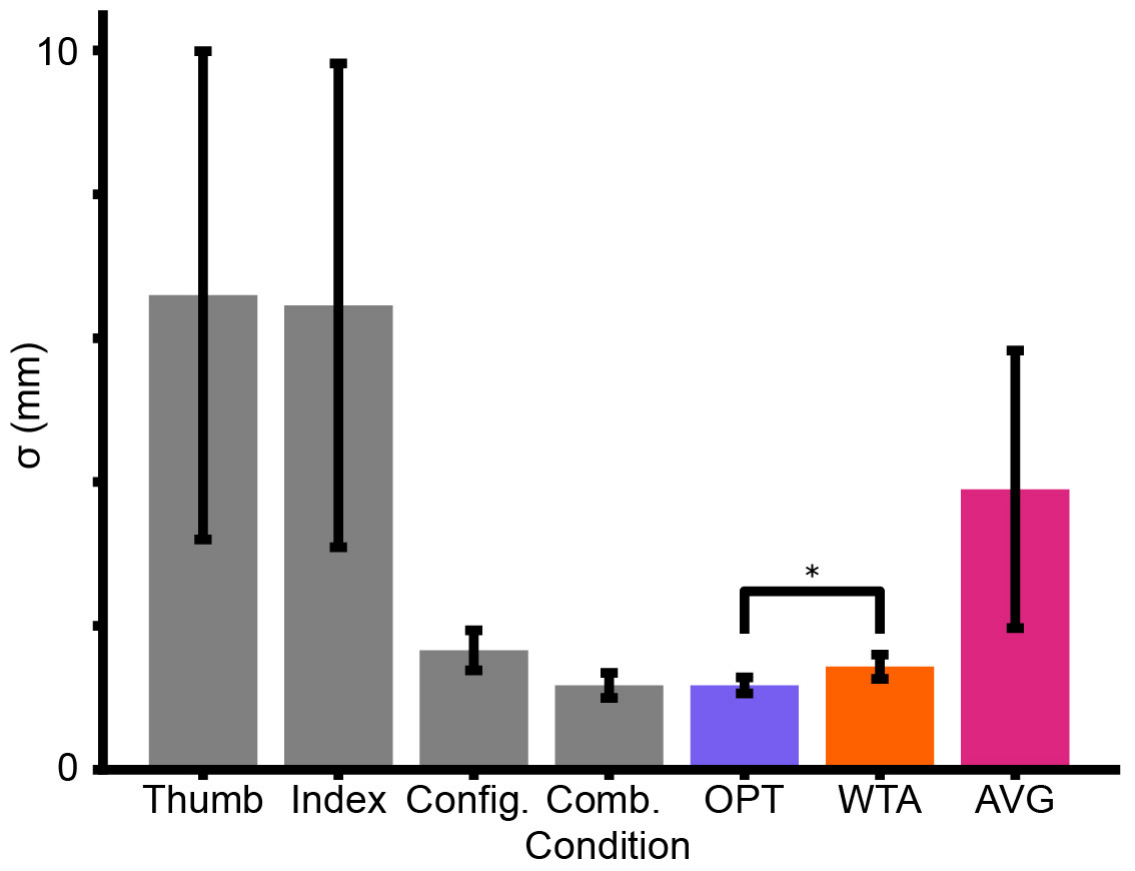
Mean sigma estimates across participants in Experiment 2 (gray bars). Mean model predictions (colored bars) for performance in the Combined Condition, based on the *Thumb, Index*, and *Config* Conditions. Error bars = ± SE. Asterisk (*) = Significant *t*-test. Note the difference in *y*-axis scale from Figure 6.

Figure 7 shows a reduction in sigma in the Cutaneous Conditions. The reliabilities of the cues in the Cutaneous and Configuration Conditions are relatively closer together and accordingly we begin to observe a greater difference between the predictions of the *OPT* and *WTA* models. One-way repeated measures ANOVAs revealed non-significant effects of condition [*p* = 0.104, *F*_(3, 30)_ = 2.236] and model prediction [*p* = 0.187, *F*_(2, 20)_ = 1.825]. Nevertheless, our planned comparison between *OPT* and *WTA* model predictions revealed a significant paired-samples *t*-test [*p* = 0.004, *t*_(10)_ = 3.740, Cohen's *d* = 0.552]. Most intriguingly, close inspection of the Figure reveals that the mean performance of participants in the Combined Condition was once again nearly identical to the *OPT* model prediction and clearly tended to be better (though not significantly so) than the *WTA* model prediction.

Single-participant Bayesian model comparison provided additional nuance and context to the above findings. The individual posterior probabilities for each participant in Experiment 2 are reported in Table 5. Bayesian model comparison categorized eight of the 11 participants as *OPT*, 2 as *AVG*, and 1 as *WTA*. While the posterior probabilities for *OPT* and *WTA* were often close, the posterior probabilities from Experiment 2 were generally more strongly in favor of *OPT* over *WTA* than were the posterior probabilities from Experiment 1. For the eight participants categorized as *OPT*, the mean posterior probabilities of *OPT* and *WTA* were 0.61 and 0.34, respectively, indicating increased confidence in the classification.

**Table 5:** Experiment 2 model posterior probabilities by participant.

**Participant**	**OPT**	**WTA**	**AVG**	**Classification**
1	0.500	0.482	0.019	OPT
2	0.188	0.385	0.427	AVG
3	0.010	0.659	0.331	WTA
4	0.199	0.370	0.430	AVG
5	0.838	0.131	0.031	OPT
6	0.455	0.437	0.109	OPT
7	0.773	0.044	0.183	OPT
8	0.538	0.462	0.000	OPT
9	0.720	0.269	0.011	OPT
10	0.502	0.498	0.000	OPT
11	0.524	0.362	0.114	OPT

### 3.3 Experiment 3

**Figure 8:**
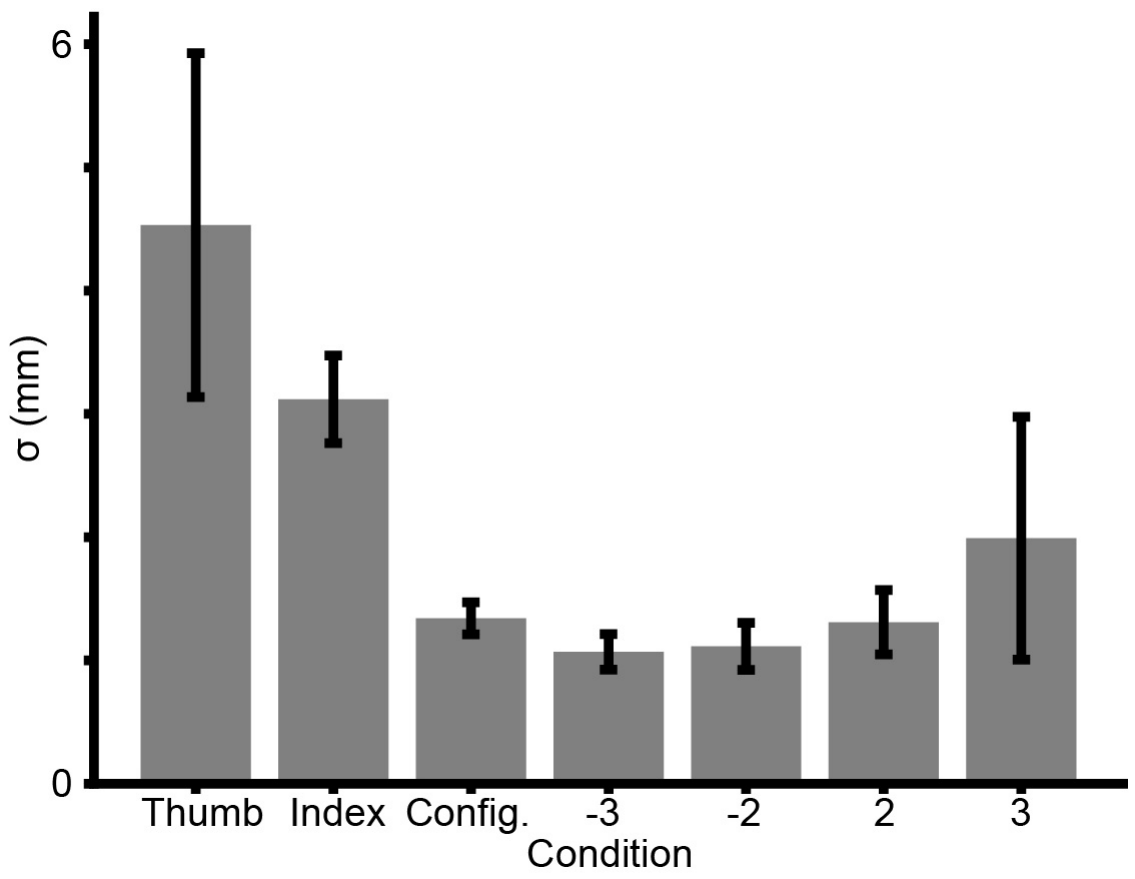
Mean sigma estimates across participants in Experiment 3 for the *Thumb, Index, Config*, and four Conflict Conditions. Error bars = ± SE.

For Experiment 3, we incorporated a cue-conflict paradigm. Figure 8 shows an overview of participant performance in each experimental condition. Observing the mean sigma estimates across participants for each condition, we found similarities to earlier experiments. The mean sigma of the cues decreased from *Thumb* to *Index* to *Config*. The four Cue Conflict Conditions are analogous to the earlier Combined Conditions of previous experiments because participants have access to all cues. As an aside, sigmas in the Conflict Conditions generally appear to be lower than the sigmas of any individual cue, with the exception of the particularly noisy **+3** Condition. Nevertheless, Experiment 3 was not intended as a replication. The predictions and planned analyses were different than in the earlier experiments.

As the reference in each of the Conflict Conditions is one of four conflict disks, each model predicts that the *PSE* should shift linearly in the direction of the cue(s) upon which the participant most relies. For example, if a participant is relying solely on *Config*, a **+3** conflict disk would feel subjectively equal to a 13 mm radius disk. Since, in a *2IFC* task, the response is to which disk felt larger, the participant would be guessing when comparing the **+3** conflict disk to a 13 mm comparison disk. On average, the proportion of times one disk is reported to be larger than the other would be 0.5: a rightward shift of 3 mm in the *PSE*.

Importantly, as is apparent from Equations 1–3, the three models predicted different linear *PSE* shifts as a function of Conflict Condition. The *WTA* model predicted a slope of +1 for any participant for whom *Config* was best. The *AVG* model predicted a slope of –1/3, because of the influence of both *Thumb* cue and *Index* cue, which produced measurements counter to that of *Config*. The *OPT* model predicted the slope shown in Equation 14 where *s* is the Conflict Condition and *ŝ* is the PSE.

**Figure 9:**
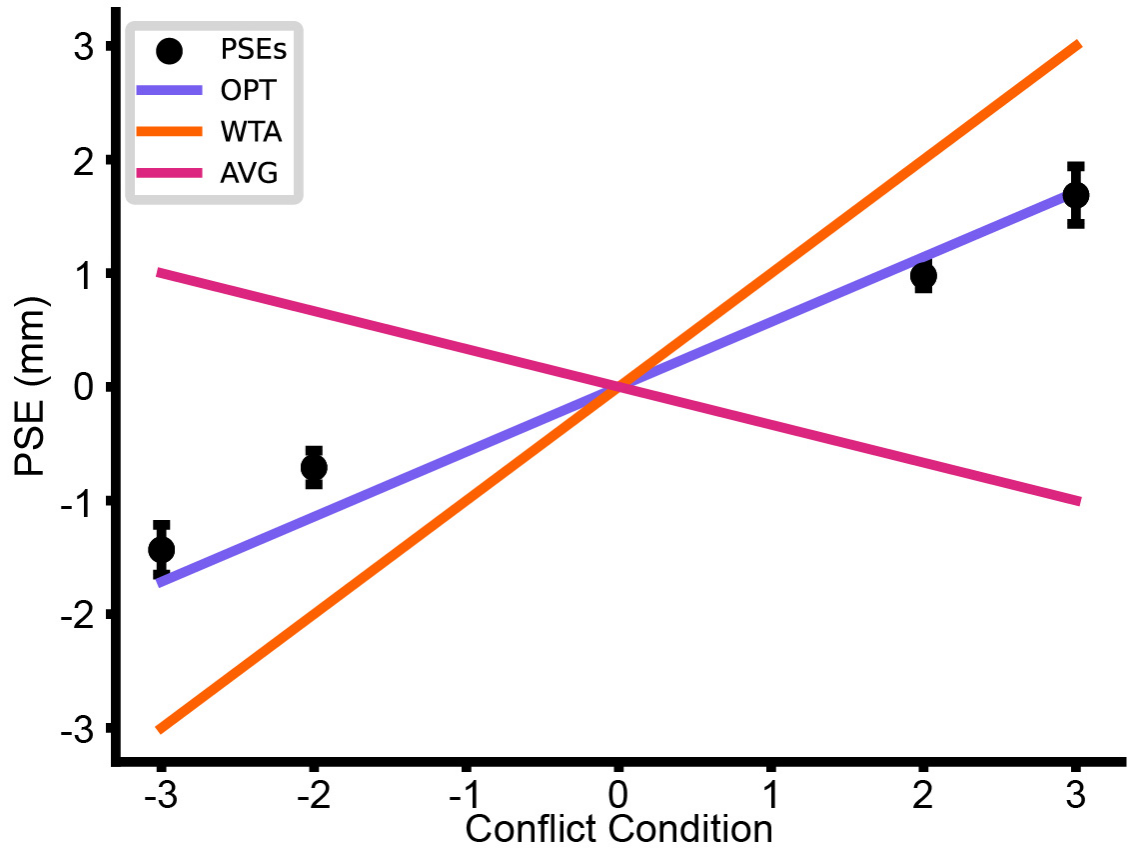
Mean PSE shift across participants by Conflict Condition (Error bars = ± SE) together with the linear relationships predicted by each of the three models.

The mean *PSE* shift across participants, as a function of Conflict Condition, is shown in Figure 9. On average, the shifting *PSEs* rather closely match the predictions of the *OPT* model. The positive slope indicates that *Config* was more heavily relied upon than *Thumb* or *Index*. Importantly, the slope is less than 1, which indicates that the *Thumb* and *Index* cues did indeed influence perception.


$$\begin{array}{l} \hat{s}=s\left(\frac{\frac{1}{\sigma_{Config}^{2}}-\frac{1}{\sigma_{Index}^{2}}-\frac{1}{\sigma_{Thumb}^{2}}}{\frac{1}{\sigma_{Thumb}^{2}}+\frac{1}{\sigma_{Index}^{2}}+\frac{1}{\sigma_{Config}^{2}}}\right) \end{array}$$ (14)

Table 6 displays the Bayesian model comparison posterior probabilities for each participant across the three models. Ten of the 11 participants were classified strongly as *OPT*, with a mean posterior probability of 0.98. Participant 11 (the one left-handed participant), in contrast, was classified strongly as *WTA*.

**Table 6:** Experiment 3 model posterior probabilities by participant.

**Participant**	**OPT**	**WTA**	**AVG**	**Classification**
1	1.000	0.000	0.000	OPT
2	1.000	0.000	0.000	OPT
3	1.000	0.000	0.000	OPT
4	0.999	0.001	0.000	OPT
5	1.000	0.000	0.000	OPT
6	0.994	0.006	0.000	OPT
7	0.999	0.000	0.001	OPT
8	0.973	0.027	0.000	OPT
9	0.871	0.001	0.127	OPT
10	1.000	0.000	0.000	OPT
11	0.000	1.000	0.000	WTA

## 4 Discussion

The present study reported three experiments that contribute increasingly supportive evidence that humans optimally combine finger configuration and cutaneous cues to perceive the size of a disk grasped between the index finger and thumb. Experiment 1 provided insight into the relative reliability of the three cues. The data suggested an *Exceptional Sigma* scenario in which *Config* was much more reliable than *Thumb* and *Index*. In such a scenario, as shown in our simulations, the *WTA* and *OPT* models make similar predictions. Accordingly, while the data from Experiment 1 provided strong evidence against the *AVG* model, we were unable to convincingly differentiate between the *WTA* and *OPT* models. In Experiment 2, we aimed to increase the reliability (lower the sigmas) of the cutaneous cues to better differentiate between the *WTA* and *OPT* models. We predicted that the use of smaller disks might result in greater skin deformation and correspondingly more reliable cutaneous cues (Goodwin et al., 1995) while leaving the configuration cue relatively unaffected. Our intention was that this would cause *OPT*, but not *WTA*, to predict a lower Combined Condition sigma, permitting us to differentiate between these two models. Experiment 2 demonstrated that reducing disk size did indeed preferentially reduce the sigmas of the cutaneous cues and enhance the difference between the predictions of the *OPT* and *WTA* models. The model comparison inferences also became more in favor of *OPT* over *WTA*. In Experiment 3, we concluded our investigation with a cue conflict experiment. By using stimuli that put the cutaneous cues in conflict with the configuration cue, we were able to glean information from *PSE* shifts. The *PSEs* showed that participants generally rely more on *Config* than *Thumb* or *Index*, but information from all cues is integrated in a way consistent with *OPT*. Our inferences in Experiment 3 were notably confident in their classifications, due to the models' distinct predictions regarding the PSE shifts. These results mirror those of our model simulations, lending credence to the conclusions. Taken together, these experiments support the conclusion that humans optimally integrate cutaneous and proprioceptive cues for haptic size perception.

### 4.1 Comparison to previous literature

Our findings are generally consistent with previous multimodal studies and (non-haptic) unimodal studies which have shown that, in a variety of perceptual tasks, participants make use of all available cues and weight these according to their reliability. Ernst and Banks (2002) found that participants could optimally combine visual and haptic cues for height. Gepshtein and Banks (2003) found participants were close to statistically optimal in a visuo-haptic size discrimination task. Alais and Burr (2004) demonstrated optimal cue combination across auditory and visual stimuli. When localizing a spatial source by a beep and a flash, participants were better with both cues than with either alone. On trials with cue conflicts, in which the beep and flash were displaced in opposite directions, participants judged the location of the event to be closer to whichever of the two cues was more reliable. Hillis et al. (2004) demonstrated optimal cue combination for two visual cues to depth: a texture cue created with Voronoi patterns and a binocular disparity cue. Negen et al. (2018) found participants, with training, could integrate a newly learned sensory skill with vision in a statistically optimal manner. In virtual reality spatial navigation and memory tasks, Newman and McNamara (2022) found that participants could combine visual landmark cues optimally, depending on task complexity and perspective. Many studies in these domains find that the reliability of cues is factored into the process of perception (Ernst and Banks, 2002; Hillis et al., 2004; Buckley and Frisby, 1993; Jacobs, 1999; van Beers et al., 2002; Gepshtein and Banks, 2003; Fetsch et al., 2009; Negen et al., 2018).

In some experimental paradigms, in non-tactile modalities such as vision, stimulus reliability can be adjusted during an experimental session. Given the need for physical stimulus delivery, this can be challenging in a tactile experiment. Through the use of virtual haptic or force-feedback devices, however, it is possible to adjust certain aspects of a haptic stimulus, such as the distance between fingers. Simultaneously adjusting a meaningful cutaneous cue, however, would be substantially more challenging. Instead, our study made use of 3D-printed disks and a calibrated mechanical system on which to mount them. We carefully selected our stimulus range to fit the limited space available. We correctly hypothesized that smaller disks would make for better cutaneous cues while leaving the finger configuration cue relatively unaffected. Logistical and engineering considerations, however, prevented us from manipulating stimulus reliability throughout the course of a single experiment.

Overall, our haptic cue combination results share the general trends of the broader literature. The consistency our results show with previous studies suggests that the brain integrates multiple tactile cues in a similar fashion as it does with other sensory modalities.

### 4.2 Future directions

Our experiments revealed some interesting examples of individual variability. For example, in Experiment 3, Participant 11, unlike the others, was more strongly *WTA* than *OPT* or *AVG*. Future experiments could examine the degree or source of such individual variability. Intriguingly, Participant 11 was the only left-handed participant. Considering that all participants used their right hand during the task, future studies could investigate the possible role of previous tactile experience (which is presumably greater with the dominant hand) in optimal cue combination. Also worthy of future study would be to investigate whether a participant who begins strongly WTA could shift toward OPT with sufficient training.

We reduced disk size to lower the cutaneous sigmas and bring their reliability closer to that of *Config*. A future study could aim to lessen the disparity between cutaneous and configuration cues by preferentially worsening *Config* and making its sigma higher. Potentially, this could be accomplished by numbing or vibrating select areas of the hand, but this manipulation would not be trivial. If such manipulations resulted in each of the three cues having *Similar Sigmas*, then the difference between the predictions of the *WTA* and *OPT* models would be at their greatest.

Future experiments could also investigate heteroskedasticity in the sensory signal. As a first approximation, our analyses assumed that participants have a single value for sigma for each cue. It is possible, however, that the trend we observed of increasing sigma, as conflict increases (Figure 8), reflects a Weber fraction (Norwich, 1987). This possibility could be investigated with experiments that use a much larger stimulus size range.

In our experiments, from one interval to another within a trial, there was a short delay between stimulus presentations. Thus, participants had to engage working memory to hold in mind the perceived size of the first disk in order to compare it to the second. Would participant performance improve, or perhaps worsen, if we were to deliver the reference to one hand and the comparison to the other, simultaneously?

Our experiments involved controlled haptics. The hand moved toward and grasped the target, but participants could not freely explore the stimuli. Future experiments could reduce the level of control in favor of a more naturalistic setting. If participants were provided unfettered access to the stimulus objects and allowed to grasp and scan the disks with all of their fingers and perhaps both of their hands, how much might performance improve? Would the *Config* cue still be most informative?

An important future research direction is to elucidate the neural mechanisms that may underlie the brain's integration of cues. Mathematically, the cue combination formulas can be understood to result from the processing of stimulus-evoked neuronal firing rates, from feature-tuned neurons that have Poisson-like firing rate noise (Ma et al., 2006). A more difficult task, and the subject of ongoing research, is to understand how such calculations are implemented by neural circuits (Pouget et al., 2003; Beck et al., 2008; Ma and Jazayeri, 2014).

Looking ahead, by contributing to the understanding of how humans combine multiple haptic cues, work such as ours may ultimately support advancements in fields such as haptic VR, robotics, and telesurgery. Our research points toward the relative importance of proprioceptive and cutaneous cues. While cutaneous cues were less reliable overall, they measurably improved performance even when finger configuration was more reliable. This highlights the importance of utilizing haptic (including cutaneous) feedback in areas like telesurgery, where incremental gains in performance can lead to invaluable improvements in outcomes. Additionally, fields such as haptic VR are constrained by the cost, size, and resolution of tactile stimulators. Cue combination research may suggest means of cleverly employing multiple cues to effectively enhance perceptual resolution within the constraints of available hardware.

## Data Availability

The raw data supporting the conclusions of this article will be made available by the authors, without undue reservation.
